# Molecular docking analysis of DNA ligase from Staphylococcus aureus with identical polysaccharides

**DOI:** 10.6026/97320630016719

**Published:** 2020-09-30

**Authors:** Anitha Roy, Aneymol Varikalam Sleeba, Rini Joy, Ponnulakshmi Rajagopal

**Affiliations:** 1 Department of Pharmacology, Saveetha Dental College and Hospital, Saveetha Institute of Medical and Technical Sciences, Chennai, Tamil Nadu, India; 2 Department of Microbiology, St. Xavier's College for Women, Aluva, Kerala-683101, India; 3 Department of Oral Medicine and Radiology, SRM Dental College and Hospitals, Ramapuram, Chennai - 600089, Tamil Nadu, India; 4 Central Research Laboratory, Meenakshi Academy of Higher Education and Research (Deemed to be Universiy) Chennai-600 078, India

**Keywords:** S.*aureus*, DNA ligase, Polysaccharide, Molecular docking

## Abstract

Staphylococcus aureus has been recognized as an important human pathogen for more than 100 years. DNA ligase is the main protein responsible for the replication of S. aureus. DNA ligase was selected as successive target to control the replication mechanism. The antibacterial activity of polysaccharide is known. Therefore, it is of interest to study the activity of Polysaccharide analogues against DNA ligase in S. aureus using molecular docking analysis. We report ten analogues using scoring parameters with best two analogues as potential drug candidate for the combat of S. aureus infection.

## Background:

Staphylococcus aureus (S. aureus) is an infective bacterium that can cause many diseases and it has become a major problem worldwide [1]. Bacterial pathogenicity involves skin and soft tissue infections, bone, joint and implant infections, pneumonia, septicemia and various toxicities such as toxic shock syndrome, scalded skin syndrome, bloodstream infections, osteomyelitis, septic arthritis and device-related infections, necrotizing fasciitis and abscesses [2]. Increased prevalence of drug resistance with bacterial pathogens, such as S. aureus has encouraged the development of strategies targeting previously untapped antibiotic mechanisms [3,4]. With an imminent crisis of antimicrobial resistance, there seems to be an urgent need to develop novel antimicrobials to fight complicated infections with multidrug resistant (MDR) pathogenic microorganisms. Desirable targets for new antimicrobials can be identified among the genes that are essential for the survival of bacteria. DNA replication is important for cell survival showing attractive targets for antimicrobials [5].

DNA ligases are the main enzyme involved in DNA repair and replication. Prokaryotic DNA ligases use NAD+ as an adenylate donor during catalysis, while eucaryotic enzymes use ATP [6]. Bacterial NAD(+)-dependent DNA ligases have also been widely studied as potential antibacterial targets due to their importance and structural differentiation from human ATP-dependent DNA ligases [7]. Therefore, it is of interest to study the activity of Polysaccharide analogues against DNA ligase in S. aureus using molecular docking analysis.

## Material and Methods:

### Target protein structure preparation:

The targeted protein DNA ligase protein (PDB ID: 3JSN) structure was downloaded from the Protein Data Bank (PDB) [8] and processed using discovery studio 2.1 The energy minimization was eventually carried out by adding the CHARMM force field.

### Ligand Preparation:

The 2-D structure of polysaccharide and its analogue structures were downloaded from pubchem database [9]. Such 2-D structures have been converted into 3-D structures using the Online Smiles Translator. H-bonds were used and the energy of the compound was reduced using the CHARMM force field. The properties of Lipinski, such as molecular weight, log P and number of hydrogen-bond donors and acceptors for active compounds, have been determined.

### Active site prediction:

Active site residues of DNA ligase were predicted using the discovery studio 2.1.

### Molecular docking:

The docking studies between the DNA ligase and polysaccharide and its analogues were performed by Lip dock in Discovery studio (Version 2.1, Accelry's Software Inc.) [10]. The active protein site was identified using the 9 Å distance volume for the site gap based on the binding site module. The Libdock method was applied to position the conformation of the compounds precisely inside the binding site. Binding score ligand poses and other score functions may be used to assess the goodness of a docking test to screen a top-ranked pose for ligands. Binding strength, hydrogen bond connections and Libdock score can be obtained in the current study and then used for final criteria.

## Results and Discussion:

**Figure 1:**
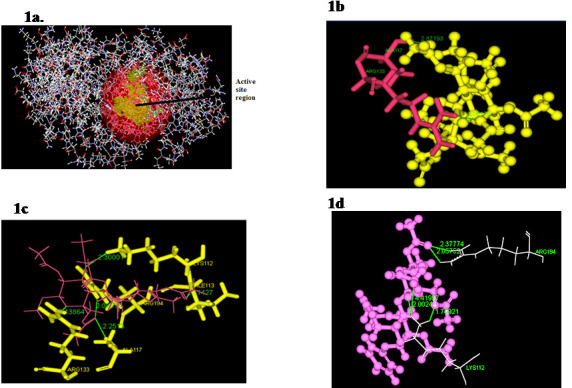
1a) Predicted site region of DNA ligase. 1 b) Molecular interaction of polysaccharide with DNA ligase. 1c) Molecular interaction of best analogues 1 with DNA ligase. 1d) Molecular interaction of best analogues 2 with DNA ligase.

The binding site is using a 9Å distance volume for site opening centered on a binding site module. The Libdock technique was then applied correctly to the position of the ligand in the active site. The process was carried out using the libdock module. The binding results show score ligand poses and multiple score parameters to calculate the effectiveness of the docking test in order to find a top-ranked pose for ligands. The binding site was defined in light red color circle as shown in Figure 1a.

Prepared compounds have been docked with target protein using the LibDock module in Discover Studio 3.2 and hundreds of pose conformations have been produced for each compound. On the basis of absolute energy, hydrogen bond interactions and Libdock ranking, the best analogs were picked. For the best conformation of the DNA ligase to the polysaccharide complex, the binding energy (136.708 kcal / mol) and the docking value (63.722) are reported. Docked complexes was visualized using discovery studio and shown in the Figure 1b, c & d. Among the selected analogues based on the scoring parameters the analogue (Pub chem id: 312811) was selected as one of the best analogue having good interaction with DNA ligase. The binding energy (133.894 kcal / mol) and docking score (63.918) were reported. The presence of hydrogen bonds is a vital criterion in identifying the binding affinity of a target with the drug for interaction. This analogue compound forms 5 H-bond interaction with LYS-112, ILE-113, ALA-117, ARG-133 & ARG-194 residues of DNA ligase protein (Figure 1c). A good receptor-ligand interaction is supported with the presence of a hydrogen bond interaction at a distance less than 3Å. Results also showed the ligand-receptor complexes with hydrogen bonding and all the bonds were within a distance less than 3 Å. The compound have a stable and a good affinity with the DNA ligase protein. Likewise yet another analogue (Pubchem ID : 266653) have the adequate binding energy of (124,962 kcal / mol) and the docking value (84,714) as shown in Table 1 (see PDF). It showed H-bond interaction through LYS-112 & ARG-194 amino acid residue (Figure 1d). We show that the amino acid residue LYS-112 & ARG-194 alternatively forms a H- bond interaction with DNA ligase protein. Thus, these two residues act as key residues for function in the target.

## Conclusion:

We report two analogues as potential drug candidates with DNA ligase for further consideration towards the combat of S. aureus infection.
